# Hybridization of Co_3_S_4_ and Graphitic Carbon Nitride Nanosheets for High-performance Nonenzymatic Sensing of H_2_O_2_

**DOI:** 10.3390/bios13010108

**Published:** 2023-01-07

**Authors:** Asha Ramesh, Ajay Ajith, Neeraja Sinha Gudipati, Siva Rama Krishna Vanjari, S. Abraham John, Vasudevanpillai Biju, Ch Subrahmanyam

**Affiliations:** 1 Department of Chemistry, Indian Institute of Technology Hyderabad, Kandi, Sangareddy 502285, Telangana, India; 2 Centre for Nanoscience and Nanotechnology, Department of Chemistry, The Gandhigram Rural Institute, Gandhigram, Dindigul 624302, Tamilnadu, India; 3 Department of Electrical Engineering, Indian Institute of Technology Hyderabad, Kandi, Sangareddy 502285, Telangana, India; 4 Research Institute for Electronic Science, Hokkaido University, Sapporo, Hokkaido 001-0020, Japan

**Keywords:** Co_3_S_4_, g-C_3_N_4_ nanosheets, H_2_O_2_ sensor, cyclic voltammetry, differential pulse voltammetry, amperometry, electrochemical sensor

## Abstract

The development of efficient H_2_O_2_ sensors is crucial because of their multiple functions inside and outside the biological system and the adverse effects that a higher concentration can cause. This work reports a highly sensitive and selective non-enzymatic electrochemical H_2_O_2_ sensor achieved through the hybridization of Co_3_S_4_ and graphitic carbon nitride nanosheets (GCNNS). The Co_3_S_4_ is synthesized via a hydrothermal method, and the bulk g-C_3_N_4_ (b-GCN) is prepared by the thermal polycondensation of melamine. The as-prepared b-GCN is exfoliated into nanosheets using solvent exfoliation, and the composite with Co_3_S_4_ is formed during nanosheet formation. Compared to the performances of pure components, the hybrid structure demonstrates excellent electroreduction towards H_2_O_2_. We investigate the H_2_O_2_-sensing performance of the composite by cyclic voltammetry, differential pulse voltammetry, and amperometry. As an amperometric sensor, the Co_3_S_4_/GCNNS exhibits high sensitivity over a broad linear range from 10 nM to 1.5 mM H_2_O_2_ with a high detection limit of 70 nM and fast response of 3 s. The excellent electrocatalytic properties of the composite strengthen its potential application as a sensor to monitor H_2_O_2_ in real samples. The remarkable enhancement of the electrocatalytic activity of the composite for H_2_O_2_ reduction is attributed to the synergistic effect between Co_3_S_4_ and GCNNS.

## 1. Introduction

H_2_O_2_ is an important chemical in many fields, including the biomedical, pharmaceutical, food, and textile industries [1,2,3]. It is also a reactive oxygen species abundant in living organisms and is essential for maintaining regular biological functions. At normal concentrations, it functions as a signaling molecule for neural development and cell proliferation and is a byproduct of cellular metabolism [4]. However, abnormal levels of H_2_O_2_ in a cell cause oxidative stress, leading to aging and diseases such as Parkinson’s, Alzheimer’s, cardiovascular diseases, cancers, or inflammation, and is, therefore, a biomarker [5,6]. As a result, the accurate and efficient determination of H_2_O_2_ is imperative, and the drive to develop inexpensive and highly sensitive H_2_O_2_ sensors has increased significantly.

There are different methods to detect H_2_O_2_**,** such as fluorimetry, titrimetry, chemiluminescence, and spectrophotometry [7,8,9,10]. However, electrochemical methods offer a better platform for fast, sensitive, inexpensive, and portable sensing [6,11]. For the electrochemical detection of H_2_O_2_, enzymatic or non-enzymatic sensors can be used. With enzymatic sensors, enzymes catalyze the reduction of H_2_O_2_. Although they have high sensitivity and good selectivity, the enzymatic electrochemical sensors suffer from the instability of the enzymes to various variables such as temperature, pH, etc., and they are expensive and have poor reproducibility [12]. Consequently, the non-enzymatic electrochemical sensing of H_2_O_2_ becomes of great importance.

The enzyme-free H_2_O_2_ sensors employ nanostructured and morphologically impressive electroactive materials, including metal oxides, sulfides, and carbon nanomaterials, as modifiers of conventional electrodes [13]. Cobalt-based nanomaterials, especially Co_3_O_4_, are extensively studied for H_2_O_2_ detection due to their good catalytic activity and high stability, and they are known to be very active towards H_2_O_2_ [1,14,15,16,17,18]. Similar in structure to Co_3_O_4_, Co_3_S_4_ is another spinel compound with superior electrochemical properties, abundant oxidation states for Faraday processes, and high theoretical specific capacity. It is mainly used for energy storage applications [19,20]. Octahedral and tetrahedral positions are occupied by cobalt in their Co^3+^ and Co^2+^ states, respectively. It is a superior electrocatalyst because the metal–sulfur bond is weaker than oxygen. Substitution with a bigger anion reduces the material’s band gap, permitting faster electron transport in Co_3_S_4_ than in Co_3_O_4_ [21,22]. However, the potential application of these materials in electrochemical sensing is not well explored, and few recent studies can be found in the literature [22,23,24,25]. The only study of Co_3_S_4_ for H_2_O_2_ detection was by Chen et al., in which they prepared core–shell Cu_2_S@Co_3_S_4_ heterostructures by hydrothermal as an electrocatalyst for H_2_O_2_ reduction. The superior electrocatalytic performance of the sensor was attributed to the microstructure and the synergistic effect between Co_3_S_4_ and Cu_2_S, where more Co(II) electrocatalytic sites are formed by the transfer of electrons from Cu_2_S to Co_3_S_4_ [24]. However, to be further explored as a sensor, the limitations of Co_3_S_4_, such as less surface area and lower conductivity, need to be improved. Hybridizing Co_3_S_4_ with high surface area and highly conductive carbon nanomaterials is one way to do this [2,16].

g-C_3_N_4_ (GCN) is a π-conjugated polymeric carbon material with a layered structure, and the C and N atoms are sp^2^ hybridized. Its structure and surface functionalities give the material good catalytic activity [26]. The bulk g-C_3_N_4_ (b-GCN) can be synthesized in good yield from low-cost materials. Due to its attractive properties and tunability, it has attracted great interest in electrochemical sensing in recent years [27,28]. The poor electrical conductivity and lower surface area of b-GCN can be significantly improved by tailoring their morphology by converting them into nanosheets. The thermal or solvent exfoliation of b-GCN yields nanosheets with high surface area and high electrical conductivity [26,29]. Ajay et al. compared the electrochemical performance of GCN nanosheets (GCNNS) prepared by solvent and thermal exfoliation and reported the superior electrocatalytic activity of solvent-exfoliated nanosheets [30]. Coupling an electroactive material with GCNNS has proven to be an excellent way to improve sensor performance [3]. In this aspect, Liu et al. integrated ZnO into the GCN matrix to form a highly sensitive H_2_O_2_ sensor. The performance enhancement of the composite resulted from increases in effective surface area and conductivity [31]. Later Atacan et al. hybridized CuO and GCN to achieve improved electrooxidation of H_2_O_2_ [32]. Ye et al. developed spherical ZnFe_2_O_4_/GCN nano-micro composites for highly efficient H_2_O_2_ sensors. The internal synergy between ZnFe_2_O_4_ and GCN promotes conductivity and improves the reaction kinetics at the electrode surface [3]. These studies emphasize the need for detailed investigations of metal oxides or sulfides hybridized with GCN as electrode materials to enhance H_2_O_2_ sensing.

In the present work, a composite is developed through hybridization between Co_3_S_4_ and GCNNS to obtain a highly sensitive and selective H_2_O_2_ sensor that works in a wide range of analyte concentrations and has a detection limit of 70 nM. Co_3_S_4_ micro flowers were synthesized by a facile hydrothermal method, b-GCN by thermal polycondensation of melamine, and the GCNNS and composite were prepared by solvent exfoliation. The electrocatalytic reduction of H_2_O_2_ was evaluated using cyclic voltammetry (CV), differential pulse voltammetry (DPV), and amperometry. As shown by impedance spectroscopy, the Co_3_S_4_/GCNNS showed a lower charge transfer resistance than its counterparts, indicating the increased electron transfer kinetics at the composite electrode due to the collective effect between Co_3_S_4_ and GCNNS. The practical application of the developed sensor was evaluated by estimating H_2_O_2_ in the human serum.

## 2. Experimental Section

### 2.1. Synthesis

#### 2.1.1. Co_3_S_4_

Co_3_S_4_ micro flowers were synthesized by hydrothermal route [33]. First, 6 mmol of cobalt (II) nitrate hexahydrate was dissolved in 60 mL of distilled water. After stirring for 10 min, 8 mmol of thiourea was added and vigorously stirred for another 15 min. Then, 4 mL of ethylenediamine was added to the above solution, and the color of the solution changed to brown. The solution was transferred to a 100 mL Teflon-lined autoclave and held at 200 °C for 12 hrs. After that, the reaction mixture was cooled to room temperature. A black-colored product was obtained, which was washed three times with distilled water and ethanol. The product was dried at 60 °C overnight.

#### 2.1.2. GCNNS

b-GCN was synthesized via the thermal polycondensation of melamine. 4 g of melamine was heated at 600 °C in a crucible for 2 hrs at 25 °C min^−1^ to obtain a yellow-colored b-GCN. The nanosheets were prepared by the solvent exfoliation of b-GCN. 10 mg of b-GCN was dispersed in 10 mL of distilled water and sonicated at 40 kHz for 2 hrs to obtain the nanosheets [30].

#### 2.1.3. Co_3_S_4_/GCNNS

First, 10 mg b-GCN was dispersed in 10 mL of distilled water and sonicated for 2 hrs at 40 kHz to obtain the nanosheets. Then, 2 mg of Co_3_S_4_ was added to the above dispersion and was further sonicated for 45 min to obtain the Co_3_S_4_/GCNNS [34,35].

### 2.2. Fabrication of the Electrodes

**Figure 1:**
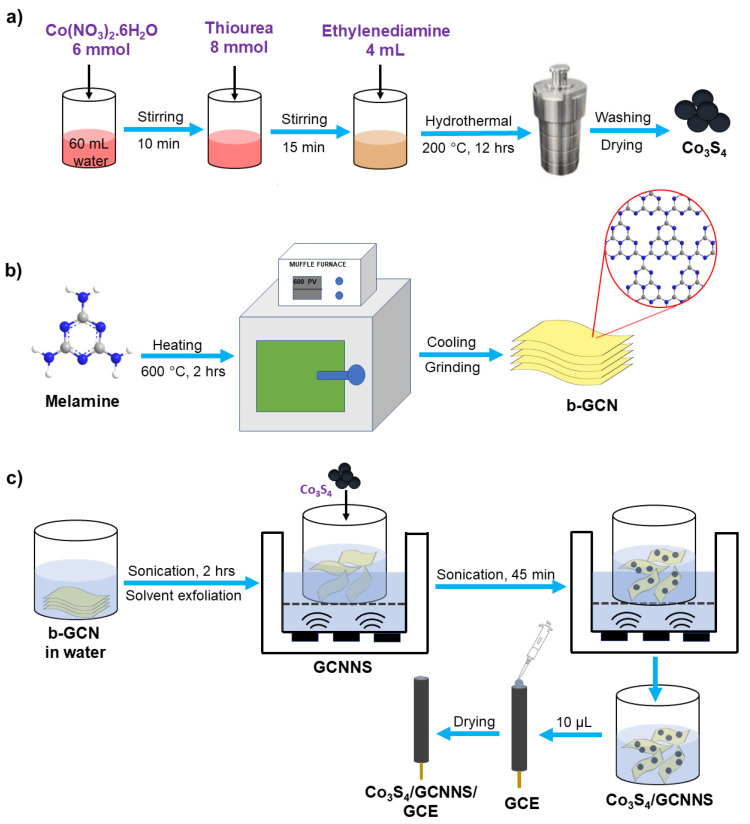
A scheme for (**a**) hydrothermal synthesis of Co_3_S_4_, (**b**) synthesis of b-GCN, and (**c**) preparation of Co_3_S_4_/GCNNS and fabrication of the electrode.

The glassy carbon electrode (GCE) was polished with 0.3 and 0.05 µm alumina slurry using a polishing cloth, sonicated for 3 min in distilled water and dried at room temperature. Drop-casting is a simple, efficient, and fast method to modify electrode surfaces [36]. 10 µL of the prepared composite was drop-cast onto the mirror-polished GCE and dried overnight at room temperature. The fabricated Co_3_S_4_/GCNNS-modified electrode was washed with distilled water and used for electrochemical studies. GCNNS-modified GCE and Co_3_S_4_-modified GCE were prepared by a similar procedure. A scheme for synthesizing the materials and the fabrication of Co_3_S_4/_GCNNS is shown in Figure 1.

## 3. Results and Discussion

### 3.1. Characterization

#### 3.1.1. Crystallographic Studies

**Figure 2:**
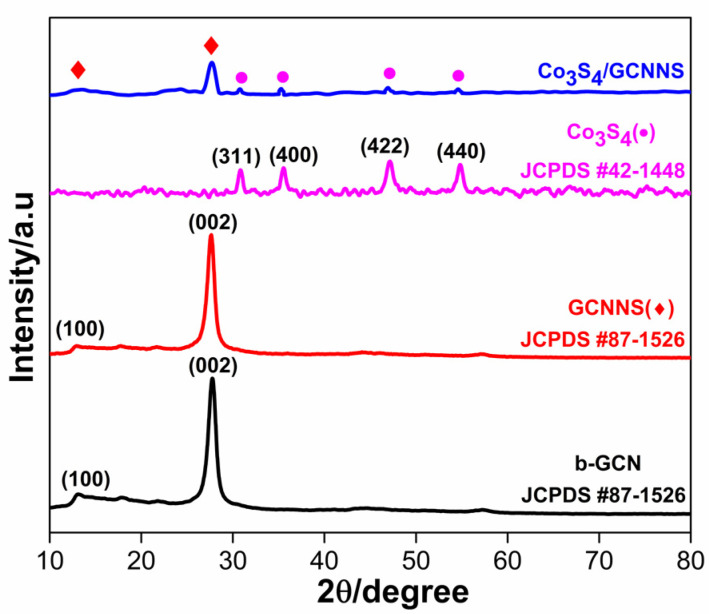
XRD data of b-GCN, GCNNS, Co_3_S_4_, and Co_3_S_4_/GCNNS.

Powder X-ray diffraction (XRD) studies of the as-prepared Co_3_S_4_, b-GCN, GCNNS, and Co_3_S_4_/GCNNS (Figure 2) help reveal the crystal and phase structure of the prepared materials. The XRD spectra of b-GCN and GCNNS are identical, and the characteristic peaks exist at 2θ values of 12.87° and 27.58° due to diffraction from (100) and (002) crystallographic plane and agree well with the standard JCPDS card no# 87-1526. The peak centered at 12.87° is a feature of the repeating tris triazine structural motifs present in b-GCN and GCNNS, and the peak at 27.58° ascribes to the periodic stacking of layers along the c-axis. The XRD spectrum of Co_3_S_4_ shows a cubic phase with the diffraction peaks at 2θ values of 31.1°, 36.04°, 47.13°, and 55.0° corresponding to the crystal planes of (311), (400), (422), and (440) aligning well with standard JCPDS card no# 42-1448. As depicted in Figure 2, the XRD pattern of the Co_3_S_4_/GCNNS nanocomposite shows the presence of both GCNNS and Co_3_S_4_, ensuring the successful formation of the composite. XRD analysis validated the synthesis of the materials, crystalline behavior, and phase purity.

#### 3.1.2. XPS Analysis

**Figure 3:**
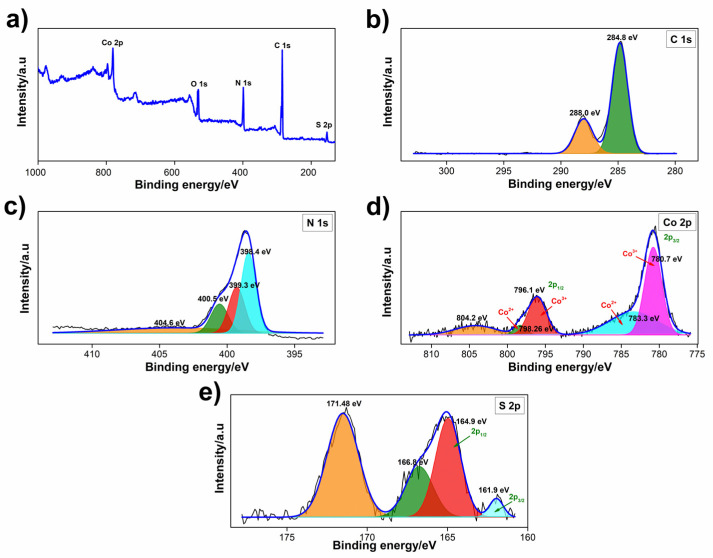
XPS spectra of Co_3_S_4_/GCNNS (**a**) survey scan, and high-resolution spectra of (**b**) C 1s, (**c**) N 1s, (**d**) Co 2p, (**e**) S 2p.

XPS studies were performed to acquire information about the chemical states of the elements in Co_3_S_4_, b-GCN, GCNNS, and Co_3_S_4_/GCNNS. The C/N atomic ratio in the b-GCN was determined to be 0.77, with the atomic percentages of C 1s and N 1s being 44.6 and 55.4, respectively. This confirms the graphitic nature of the prepared b-GCN as it contains C and N in a nearly 3:4 ratio [37,38]. The spectra of Co_3_S_4_/GCNNS are shown in Figure 3, and those of GCNNS and Co_3_S_4_ are in Figure S1 (Supplementary Materials). The full-scan spectrum of Co_3_S_4_/GCNNS in Figure 3a reveals the presence of C, N, Co, and S in the composite. The O detected is due to the oxidation or absorption of oxygen by the sample in the air [39]. As shown in Figure 3b, the high-resolution spectra of C 1s can be deconvoluted into two peaks at 284.8 and 288.0 eV. The peak at 284.8 eV is attributed to graphitic or amorphous carbon present in GCNNS or adsorbed on the surface. The carbon atoms in the $N=C-{\left(N\right)}_{2}$ group in GCNNS yield a peak at 288.0 eV [35,40]. The high-resolution spectrum of N 1s (Figure 3c) combines four peaks at 398.4, 399.3, 400.5, and 404.6 eV. The peak at 398.4 eV is the N sp^2^ bond in C−N=C in the triazine ring, and the peaks at 399.3 and 400.5 eV correspond to the tertiary nitrogen group $\left(N-{\left(C\right)}_{3}\right)$ and the quaternary N three-carbon atom amino functional group (N−H) in the aromatic ring, respectively. The peak at 404.6 eV corresponds to the π excitation of C=N in GCNNS [41].

The high-resolution spectra of Co 2p in the composite (Figure 3d) can be deconvoluted into two spin-orbit doublets of Co^2+^ and Co^3+^. The peaks 14.96 eV apart at 783.3 and 798.26 eV correspond to the 2p^3/2^ and 2p^1/2^ orbitals of Co^2+^ in Co_3_S_4_. Whereas the 2p^3/2^ and 2p^1/2^ doublet of Co^3+^ appears at 780.7 and 796.1 eV with a spacing of 15.4 eV, suggesting the existence of Co in the +2 and +3 oxidation states in Co_3_S_4_/GCNNS and a satellite peak appears at 804.2 eV [42,43]. The S 2p spectra (Figure 3e) of Co_3_S_4_/GCNNS are fitted into four peaks, the peaks at 161.9 and 164.9 eV are indexed to S 2p^3/2^ and S 2p^1/2^ of S in Co_3_S_4_ and the two peaks at 166.8 and 171.48 eV belong to S in ${\mathrm{SO}}_{3}^{2-}$ and ${\mathrm{SO}}_{4}^{2-}$, respectively [41]. The results demonstrate the successful formation of the Co_3_S_4_/GCNNS composite. As shown in Figure S1a, the survey scan of GCNNS showed the presence of C 1s and N 1s, while Co_3_S_4_ showed the presence of Co 2p and S 2p, and the deconvoluted spectra are shown in Figure S1b,c,e,f. The spectral peaks of the materials are presented in Table S1 for comparison. Interestingly, the binding energy of Co 2p has increased in the composite compared to pure Co_3_S_4_, and the binding energy of C 1s has decreased compared to its pure counterpart, strongly suggesting the electron transfer between Co_3_S_4_ and GCNNS in the composite. The decrease in electron density of Co_3_S_4_ in the composite causes an increase in its binding energy, attributed to the transfer of electrons from Co_3_S_4_ to GCNNS in the hybrid structure. Meanwhile, the electronegativity of Co (1.88) is lower than that of C and N, further proving that the electron of Co_3_S_4_ tends to donate to GCNNS. These results prove that the integration between Co_3_S_4_ and GCNNS in the composite is not just a physical mixture but that there is heterojunction formation with strong electronic interactions [44].

#### 3.1.3. UV-Visible

**Figure 4:**
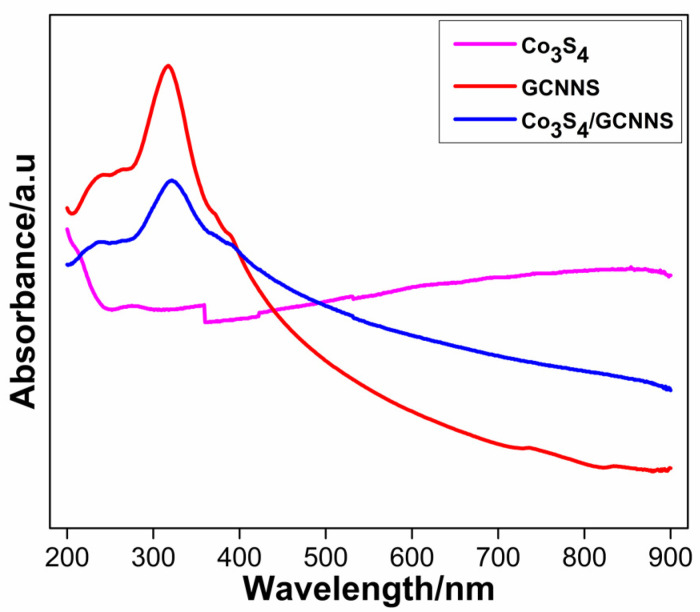
UV-visible absorption spectra of Co_3_S_4_, GCNNS and Co_3_S_4_/GCNNS.

The UV-visible spectra of Co_3_S_4_, GCNNS, and Co_3_S_4_/GCNNS are shown in Figure 4. GCNNS shows a characteristic absorption band at 318 nm due to the π-π* transition in GCNNS, as previously reported [30]. The corresponding absorption in Co_3_S_4_/GCNNS appears at 321 nm, although the intensity of the absorption has diminished. The slight wavelength shift and decrease in adsorption intensity are attributed to composite formation. Co_3_S_4_ displays a broad absorption in the 400–800 nm region. Due to the integration of Co_3_S_4_ into the GCNNS matrix, the Co_3_S_4_/GCNNS exhibits greater absorption in the visible light region than that of GCNNS.

#### 3.1.4. SEM and EDS

**Figure 5:**
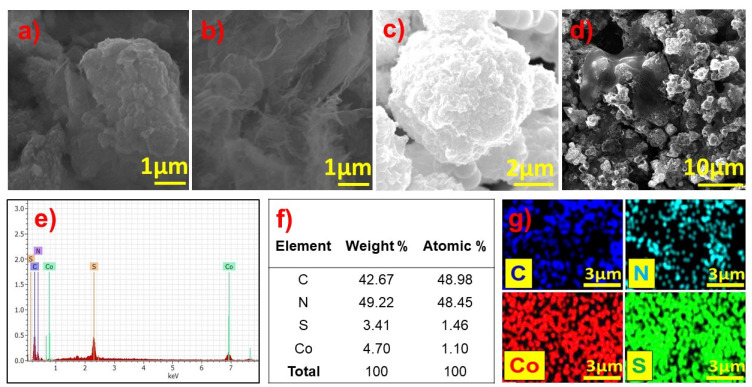
SEM images of (**a**) b-GCN, (**b**) GCNNS, (**c**) Co_3_S_4_, (**d**) Co_3_S_4_/GCNNS and (**e**) EDS spectrum of Co_3_S_4_/GCNNS, (**f**) elemental percentages in Co_3_S_4_/GCNNS and, (**g**) elemental maps of Co_3_S_4_/GCNNS.

The morphology of the synthesized materials was examined by SEM and is shown in Figure 5. The SEM micrograph of the b-GCN shown in Figure 5a is a highly aggregated structure. Further GCNNS was drop-cast and dried on a glassy carbon (GC) plate to record the SEM images (Figure 5b). Interestingly, wrinkled layers with a few-nanometer thicknesses are observed, confirming the formation of the nanosheets. The recorded SEM micrographs of Co_3_S_4_ (Figure 5c) show a micron-sized flower-like morphology consistent with the literature [33]. Co_3_S_4_ micro flowers sonicated in water were drop-cast, dried on a GC plate, and examined by SEM to understand whether structural distortion occurs under sonication. No morphological changes were observed. Furthermore, the Co_3_S_4_/GCNNS modified on the GC plate was analyzed, and as shown in Figure 5d, the composite showed both GCNNS and Co_3_S_4_ micro flowers, with Co_3_S_4_ embedded on the GCNNS surface. The Co_3_S_4_ and GCNNS retain their structure in the composite; however, slight agglomeration was observed in the composite. The elemental composition of the materials was analyzed using EDS. As shown in Figure 5e, the EDS spectrum of Co_3_S_4_/GCNNS showed the presence of C, N, Co, and S, confirming the formation of the composite. The respective atomic and weight percentages of the elements are presented in Figure 5f. The uniform distribution of the elements can be understood from the elemental mapping shown in Figure 5g.

#### 3.1.5. TEM

**Figure 6:**
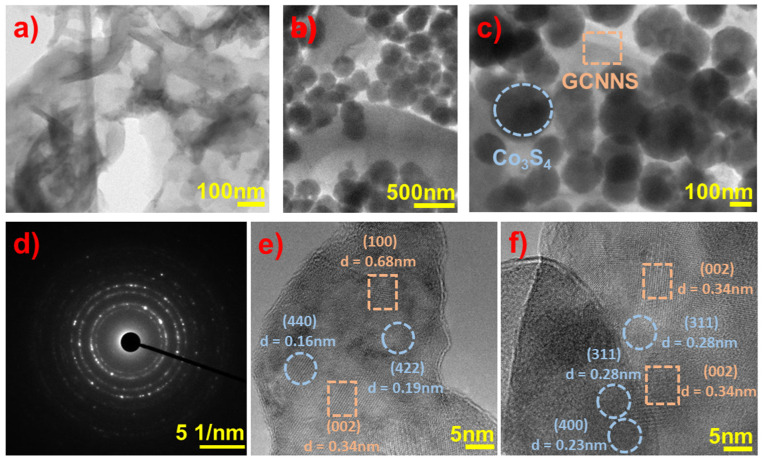
TEM images of (**a**) GCNNS, (**b**,**c**) Co_3_S_4_/GCNNS, (**d**) SAED pattern of Co_3_S_4_/GCNNS, (**e**,**f**) HRTEM images of Co_3_S_4_/GCNNS.

TEM images of GCNNS and Co_3_S_4_/GCNNS were acquired to examine the structure of the materials in detail, and the results are displayed in Figure 6. As shown in Figure 6a, GCNNS consists of very thin, wrinkled nanosheets. The lack of transparency observed in some regions is due to the presence of multilayers. TEM images of Co_3_S_4_/GCNNS acquired at different magnifications are shown in Figure 6b,c. It is observed that sphere-shaped Co_3_S_4_ are attached to the surface of GCNNS nanosheets and confirm the formation of the composite. Figure 6e,f show the high-resolution TEM (HRTEM) images captured from the Co_3_S_4_/GCNNS composite; the fringes with d spacing values of 0.28 nm, 0.23 nm, 0.19 nm, and 0.16 nm can be ascribed to the (311), (400), (422), and (440) crystallographic planes of Co_3_S_4_ (JCPDS card no# 42-1448). The GCNNS fringes with d spacing values of 0.68 nm and 0.34 nm correspond to the (100) and (002) planes (JCPDS card no# 87-1526). It can be observed that the Co_3_S_4_ fringes are closely surrounded by GCNNS fringes in the composite, indicating the strong interfacial contact between the materials, which is favorable for the faster electron transfer to promote the electrocatalytic reduction of H_2_O_2_. As shown in Figure 6d, the SAED pattern of Co_3_S_4_/GCNNS has a concentric ring structure, revealing the polycrystalline nature of the composite. TEM analysis confirms the successful coupling of GCNNS and Co_3_S_4_ in the composite.

### 3.2. Electrochemical Characterization of Modified Electrodes

#### 3.2.1. Response of Co_3_S_4_ and Co_3_S_4_/GCNNS Modified GCE Electrode in NaOH

**Figure 7:**
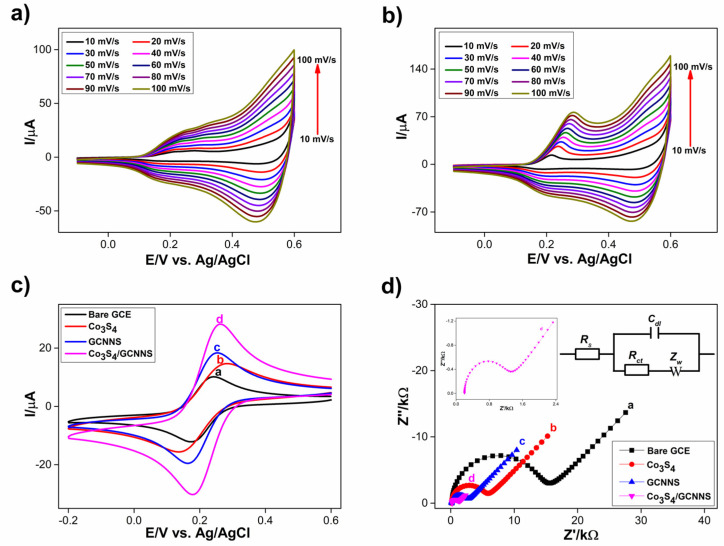
CVs obtained for the response of electrode in 0.1 M NaOH at different scan rates from 10–100 mV s^−1^ (**a**) at the Co_3_S_4_, (**b**) at the Co_3_S_4_/GCNNS, (**c**) CVs obtained for the response of modified electrodes in K_4_[Fe(CN)_6_]/K_3_[Fe(CN)_6_] and (**d**) Nyquist plots of electrodes obtained in K_4_[Fe (CN)_6_]/K_3_[Fe(CN)_6_] at (**a**) bare GCE (**b**) Co_3_S_4_, (**c**) GCNNS and (**d**) Co_3_S_4_/GCNNS-modified GCEs.

The formation of the Co_3_S_4_ and Co_3_S_4_/GCNNS layer on the GCE surface was examined by recording cyclic voltammograms of the modified electrodes in 0.1 M NaOH at different scan rates of 10–100 mV/s and is shown in Figure 7a and Figure 7b, respectively. The cyclic voltammograms of Co_3_S_4_ show four characteristic peaks, including two anodic peaks at 0.22, and 0.50 V and two cathodic peaks at 0.20 and 0.51 V, as shown in Figure 6a, and are consistent with the literature [15,45]. As the scan rate increases, the peak currents increase. These peaks arise from reversible electrochemical redox reactions of cobalt in different oxidation states in the Co_3_S_4_. Although the mechanisms of these reactions in Co_3_S_4_ are not well understood, they are expected to be similar to the well-reported redox reactions of Co(OH)_2_ since the CV profile is similar. There is only a slight deviation in the anodic and cathodic peak potentials of Co_3_S_4_ from that of Co(OH)_2_. Oxygen and sulfur also belong to the same group [46]. Furthermore, GCE modified with Co_3_S_4_/GCNNS (0.2 mg/mL) was characterized in the same way, as illustrated in Figure 6b. The corresponding anodic peak observed in Co_3_S_4_ can also be seen in the composite in the positive potential scan, and there is an increase in the peak currents, and the peak at 0.22 V becomes prominent. During the negative potential scan, two cathodic peaks corresponding to that in the pure Co_3_S_4_ are visible, and the reduction peak currents have increased. The results confirm the presence of cobalt in both Co_3_S_4_ and Co_3_S_4_/GCNNS electrodes and suggest an improvement in the redox activity of the Co_3_S_4_ by the composite formation with GCNNS.

#### 3.2.2. K_4_[Fe(CN)_6_]/K_3_[Fe(CN)_6_] Response of Modified Electrodes and Electroactive Surface Area (EAS)

The electrochemical behavior of bare GCE, Co_3_S_4_, GCNNS, and Co_3_S_4_/GCNNS-modified GCE was investigated with K_4_[Fe(CN)_6_] and K_3_[Fe(CN)_6_] redox probes. Cyclic voltammograms of the modified electrodes were recorded in 0.1 M KCl containing 1 mM of K_4_[Fe(CN)_6_]/K_3_[Fe(CN)_6_] each, from a potential range of 0.6 to −0.2 V vs. Ag/AgCl at a scan rate of 50mV/s and are shown in Figure 7c. A well-defined reversible redox peak for the K_4_[Fe(CN)_6_]/K_3_[Fe(CN)_6_] system is observed. Bare GCE showed an oxidation peak current of 10.2 µA, and the redox peaks are separated by 67 mV (ΔE_p_). The oxidation peak current was increased when GCE was modified with Co_3_S_4_ to a value of 14.88 µA, although the ΔEp value has increased to 140 mV. Meanwhile, the GCNNS-modified electrode yielded an oxidation current of 18.57 µA, 1.25 times higher than Co_3_S_4_, and displayed a peak separation of 90 mV lower than for Co_3_S_4_. The increase in the response of GCNNS to Fe^2+^/Fe^3+^ is because the nanosheet morphology of GCNNS favors electron transfer between the analyte and the electrode. These results illustrate the superior electrocatalytic activity of GCNNS towards the Fe^2+^/Fe^3+^ couple compared to Co_3_S_4_. The Co_3_S_4_/GCNNS-modified GCE showed a maximum peak current of 28.20 µA, 1.89 and 1.51 times higher than Co_3_S_4_ and GCNNS with a ΔEp of 82 mV. Interestingly, the hybridization of Co_3_S_4_ with GCNNS resulted in a significant increase in the oxidation peak current and a reduced ΔEp compared to its counterparts, strongly indicating the enhanced electrocatalytic aspects and the importance of the synergistic effects of the composite. The electroactive surface area (EAS) of the fabricated electrodes was calculated using the Randles–Sevcik Equation (1),

(1) $$I_{p}=2.69\times 10^{5}n^{3/2}AD^{1/2}C\nu^{1/2}$$

where *I_p_* is the peak current at the respective electrode, *n* is the number of electrons involved (1 for [Fe(CN)_6_]^3−/4−^), *A* is the EAS of the electrode in cm^2^, *D* is the diffusion coefficient (6.7 × 10^6^ cm^2^/s), *C* is the concentration of the redox couple (1 mM), and *ν* is the scan rate (50 mV/s). The EAS of GCE, Co_3_S_4_, GCNNS, and Co_3_S_4_/GCNNS-modified GCE was estimated to be 0.067, 0.097, 0.12, and 0.18 cm^2^, respectively. The Co_3_S_4_/GCNNS-modified GCE has the highest EAS, 1.9 times higher than Co_3_S_4_ and 1.5 times higher than GCNNS which gives the composite electrode high electrocatalytic activity compared to their pure counterparts.

#### 3.2.3. Electrochemical Impedance Studies

The impedance changes occurring at the electrode surface were further investigated using electrochemical impedance spectroscopy. Nyquist plots of bare GCE, Co_3_S_4_, GCNNS, and Co_3_S_4_/GCNNS-modified GCE electrodes were recorded in 0.1 M KCl containing 1 mM K_4_[Fe(CN)_6_]/K_3_[Fe(CN)_6_], and the obtained plots are shown in Figure 7d. The impedance data were simulated using the Randles equivalent circuit model and are shown in Figure 7d (inset), where *R_S_* is the ohmic resistance of the electrolyte, *R_CT_* is the charge transfer resistance, *C_dl_* is the double layer capacitance, and *Z_W_* is the Warburg impedance. The semi-circular region in the Nyquist plots corresponds to the electron transfer limited process, and the diameter is equal to the *R_CT_*. The *R_CT_* values of the electrodes were determined to be 15.80, 6.28, 2.88, and 1.40 kΩ for bare, Co_3_S_4_, GCNNS, and Co_3_S_4_/GCNNS modified GCEs, respectively. When modified with Co_3_S_4_ or GCNNS, the *R_CT_* values become lower than bare GCE values. Meanwhile, Co_3_S_4_/GCNNS has the lowest *R_CT_* value, demonstrating fast electron transfer mediated by the composite, compared to the pure counterparts. The heterogeneous electron transfer rate constant (*k_et_*) of K_4_[Fe(CN)_6_]/K_3_[Fe(CN)_6_] at the modified electrode is calculated using Equation (2),

(2) $$k_{et}=RT/n^{2}F^{2}AR_{CT}C^{0}$$

where *R* is the gas constant (8.314 J.mol^−1^.K^−1^), *T* is the temperature (298 K), *n* is the number of electrons transferred per molecules of the redox probe (*n* = 1 for the [Fe(CN)_6_]^3−/4−^), *F* is the Faraday constant (96,485 C), *A* is the area of the electrode (0.07 cm^2^), *R_CT_* is the charge transfer resistance at the respective electrode, and *C^0^* is the concentration of the redox couple in the bulk solution (1 mM). The *k_et_* of GCE, Co_3_S_4_, GCNNS, and Co_3_S_4_/GCNNS-modified composite electrodes were found to be 2.37 × 10^−4^, 5.97 × 10^−4^, 13.03 × 10^−4^, and 26.71 × 10^−4^ cm^2^ s^−1^, respectively. The high *k_et_* value achieved by Co_3_S_4_/GCNNS highlights the facile and faster electron transfer reaction at this electrode surface than at Co_3_S_4_ or GCNNS. The results confirm that an efficient electrical network through Co_3_S_4_ anchored on the surface of GCNNS facilitates electron transfer in the composite-modified GCE. To further investigate the charge transfer, the photoluminescence (PL) of the materials was measured and is shown in Figure S2. At an excitation wavelength of 330 nm, both GCNNS and Co_3_S_4_/GCNNS show PL peaks around 450 nm. The GCNNS showed the highest PL intensity, indicating the faster recombination of photogenerated e^-^ and h^+^ [38]. The decrease in PL intensity in the composite is due to the quenching of carrier recombination by Co_3_S_4_, and these electrons can be transferred at the interface. The results indicate that the heterogeneous electron transfer rate in Co_3_S_4_/GCNNS is larger than in GCNNS and is beneficial for H_2_O_2_ sensing.

### 3.3. Electrochemical Detection of H_2_O_2_

#### 3.3.1. Electrochemical Reduction of H_2_O_2_ at Co_3_S_4_/GCNNS Modified GCE

**Figure 8:**
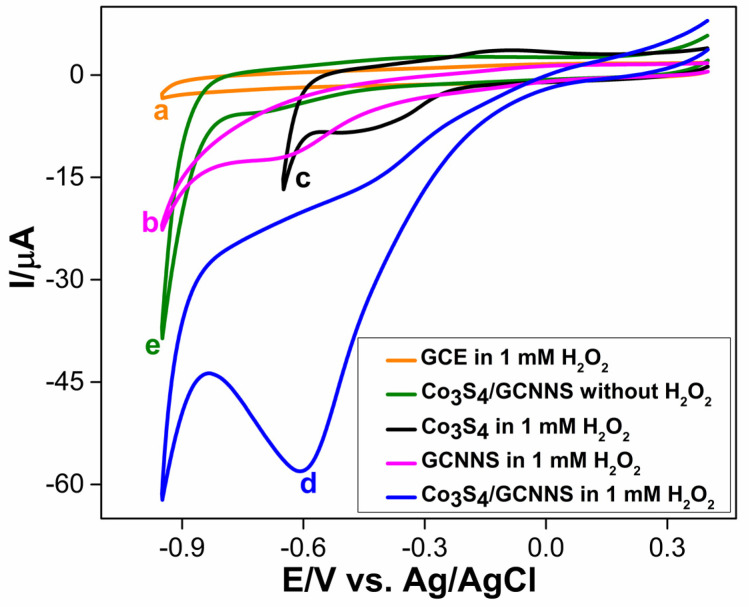
CVs obtained for the reduction of 1 mM H_2_O_2_ at (**a**) bare GCE, (**b**) GCNNS, (**c**) Co_3_S_4_, (**d**) Co_3_S_4_/GCNNS and (**e**) at Co_3_S_4_/GCNNS in the absence of H_2_O_2_, in 0.2 M PBS (pH 7.2) at a scan rate of 50 mV/s.

The electrocatalytic properties of the fabricated electrodes towards H_2_O_2_ reduction were initially investigated using CV in a 0.2 M phosphate-buffered solution (PBS) of pH 7.2. Figure 8 shows the cyclic voltammograms obtained for bare and modified electrodes in the absence or presence of 1 mM H_2_O_2_, recorded between a potential range of 0.4 to −0.95 V at a scan rate of 50 mV/s. A bare GCE shows no redox features in the presence of H_2_O_2_ in Figure 8 (curve a). Figure 8 (curve b) shows that the GCE modified with GCNNS reduces H_2_O_2_ at a potential of −0.65 V to yield a peak current of −12.21 µA. The amino or cyano groups caused the activity on the surface of the GCNNS. The abundance of N atoms with lone pairs in their sp^2^ orbitals in the GCNNS also aids in the adsorption of tiny molecules, such as H_2_O_2_ [27,32]. In this case, this favorable interaction between the analyte and the material facilitates electrocatalytic reduction. On the other hand, the electrochemical reduction of H_2_O_2_ at the Co_3_S_4_-modified GCE has the advantage of a lower reduction potential, as shown by Figure 8 (curve c). H_2_O_2_ is reduced at 200 mV less negative potential than the GCNNS electrode, but the peak current is less (−8.14 μA).

At the Co_3_S_4_/GCNNS electrode, a sharp and enhanced reduction peak can be observed at a potential of −0.6 V for the electroreduction of H_2_O_2_ in Figure 8 (curve d), while the electrode shows no response in the absence of H_2_O_2_, as seen in Figure 8 (curve e). The composite electrode delivers a large current of −58.2 μA, 4.7 times higher and 7.1 times higher than GCNNS and Co_3_S_4_-modified GCEs, respectively. Interestingly, the H_2_O_2_ reduction potential at Co_3_S_4_/GCNNS is reduced to 50 mV less negative than that at the GCNNS electrode. The cyclic voltammetric results show that the GCE modified with Co_3_S_4_/GCNNS shows the best performance towards H_2_O_2_ reduction.

The remarkable enhancement in the reduction peak currents can be attributed to the synergistic effect between Co_3_S_4_ and GCNNS that might have fastened the electron transfer with the H_2_O_2_ at the electrode surface. Co_3_S_4_ has good catalytic activity towards the reduction of H_2_O_2_ as it reduces the analyte at a lower potential. Still, the reduction peak current is lower, possibly due to the lower conductivity and small surface area of the Co_3_S_4_ micro flowers (0.097 cm^2^). When combined with GCNNS, the effective surface area and conductivity are improved. As discussed earlier, the Co_3_S_4_/GCNNS electrode has a high electroactive surface area (0.18 cm^2^), the lowest *R_CT_* value (1.40 kΩ), indicating improved conductivity, and the highest charge transfer constant to facilitate faster electron transfer between the electrode and the analyte. These parameters are decisive in the superior electrocatalytic activity of the Co_3_S_4_/GCNNS electrode towards H_2_O_2_ reduction. All of these observations emphasize the excellent electrocatalytic activity of the Co_3_S_4_/GCNNS electrode.

The possible mechanism for the electroreduction of H_2_O_2_ at Co_3_S_4_/GCNNS can be expressed as in Equations (3)–(8). H_2_O_2_ reduction at the composite electrode occurs via the direct two-electron transfer pathway $\left(H_{2}O_{2}+2e^{-}\to 2{\mathrm{OH}}^{-}\right)$ and is converted to H_2_O. In the composite, both GCNNS and Co_3_S_4_ involve the reduction mechanism. The reaction at the GCNNS surface can be expressed as in Equations (3)–(5), where the active sites are the nitrogen-containing functional groups. While in Co_3_S_4_, H_2_O_2_ is reduced by the Co(II) ions and is converted to Co_3_S_4_(OH). Co(III) in Co_3_S_4_(OH) is again electro-reduced to Co(II), and Co_3_S_4_ is regenerated at the electrode surface. The corresponding mechanism is shown by Equations (6)–(8).

(3) $$H_{2}O_{2}+e^{-}\to OH_{ads}+OH^{-}$$

(4) $$OH_{ads}+e^{-}\to OH^{-}$$

(5) $$OH^{-}+H^{+}\to H_{2}O$$

(6) $$2Co_{3}S_{4}+H_{2}O_{2}\to 2Co_{3}S_{4}\left(OH\right)$$

(7) $$2Co_{3}S_{4}\left(OH\right)+2e^{-}\to 2Co_{3}S_{4}+2OH^{-}$$

(8) $$OH^{-}+H^{+}\to H_{2}O$$

As indicated by XPS, there is electron transfer between GCNNS and Co_3_S_4_, which could increase the active sites available for redox reactions. Additionally, the coordination bonds formed between the cobalt ion and the lone nitrogen pairs stabilize Co_3_S_4_ on the GCNNS surface, and since GCNNS readily adsorbs H_2_O_2_ on its surface, this also increases the interaction of H_2_O_2_ with Co(II) active sites and thereby favors a stronger reduction.

**Figure 9:**
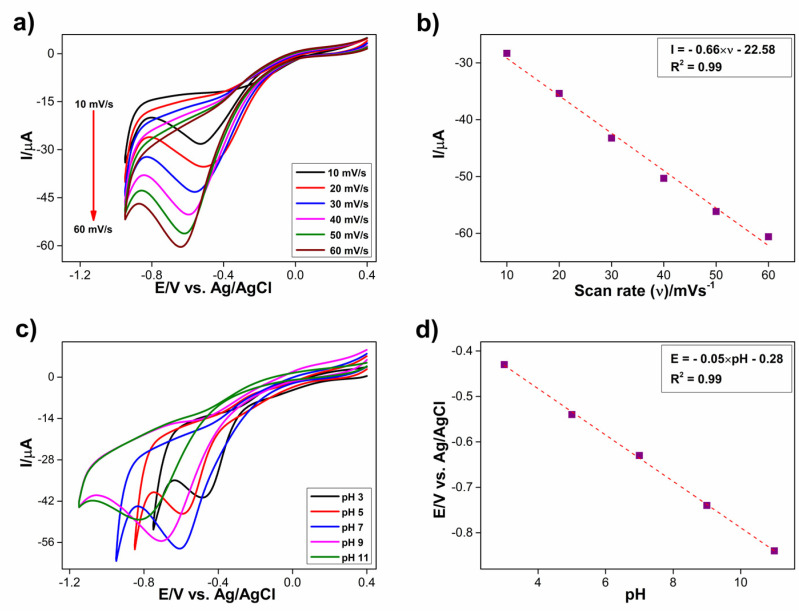
(**a**) CVs obtained for the reduction of 1 mM H_2_O_2_ at Co_3_S_4_/GCNNS in 0.2 M PBS at different scan rates from 10–60 mV s^−1^, (**b**) calibration plot of peak current with respect to the scan rate, (**c**) CVs obtained for the reduction of 1 mM H_2_O_2_ at the Co_3_S_4_/GCNNS electrode at different pH of 3, 5, 7, 9, and 11 in 0.2 M PBS at a scan rate of 50 mV/s, (**d**) plot of the reduction potential of H_2_O_2_ vs. pH of the PBS.

The response of Co_3_S_4_/GCNNS to 1 mM H_2_O_2_ was further studied by varying the scan rate from 10 to 60 mV/s, as shown in Figure 9a. Upon increasing the scan rates, the cathodic peak currents were increased. The peak current vs. scan rate was plotted, as shown in Figure 9b, and it showed a good linear relationship. The regression expression is I = −0.66 × ν – 22.58 with an R^2^ value of 0.99. This indicates that the reduction of H_2_O_2_ at the Co_3_S_4_/GCNNS electrode is a surface adsorption-controlled process. The electrochemical reduction behavior of the composite electrode towards 1 mM H_2_O_2_ in different pH ranges from 3 to 11 was studied using CV in 0.2 M PBS at a scan rate of 50 mV/s. With an increase in the pH, the reduction peak potential of H_2_O_2_ on the composite electrode shifts to more negative values, as seen in Figure 9c. The shift in the peak potentials suggests that protons are involved in the electrochemical reduction. The reduction peak currents increase upon increasing the pH from 3 to 7 and decrease thereafter. The peak current achieves the maximum at a pH of 7; hence it is chosen for the electrochemical studies to determine H_2_O_2_. The relationship of reduction peak potential vs. pH is plotted in Figure 9d, showing a linear relationship. The regression equation is E = −0.05 × pH − 0.28 with an R^2^ value of 0.99. The slope of the curve is around 59 mV, which matches the theoretical value for the two protons and two electrons reaction.

The loading levels of Co_3_S_4_ and GCNNS were varied to optimize the performance of the composite electrode. First, the concentrations of Co_3_S_4_ varied between 0.1, 0.15, 0.2, and 0.25 mg in 1 mg/mL GCNNS. The cyclic voltammograms recorded for 1 mM H_2_O_2_ are shown in Figure S3a, in which the peak current increases up to a load of 0.2 mg; beyond that, the current decreases, and the shape of the CV curve changes as the capacitance current and onset potential increase. This is possible because above 0.2 mg, Co_3_S_4_ agglomerates on the GCNNS surface, reducing the number of catalytically active sites available for H_2_O_2_ reduction.

Furthermore, the weight of GCNNS was varied to 0.5, 1, and 1.5 mg for a weight of Co_3_S_4_ of 0.2 mg/mL water, and the recorded CV curves are shown in Figure S3b. At a loading of 0.5 mg/mL, the peak reduction current is lower; with an increase in the GCNNS level, there is an increase in peak current, but above 1 mg/mL, the activity decreases. This is due to the agglomeration of the nanosheets and the loss of active sites on the electrode surface due to the increased loading. Therefore, the optimal combination to produce the best-performing Co_3_S_4_/GCNNS composite electrode was determined to be 0.2 mg Co_3_S_4_ and 1 mg GCNNS in 1 mL distilled water, which was then used for subsequent electrochemical studies.

#### 3.3.2. Sensitive Determination of H_2_O_2_

**Figure 10:**
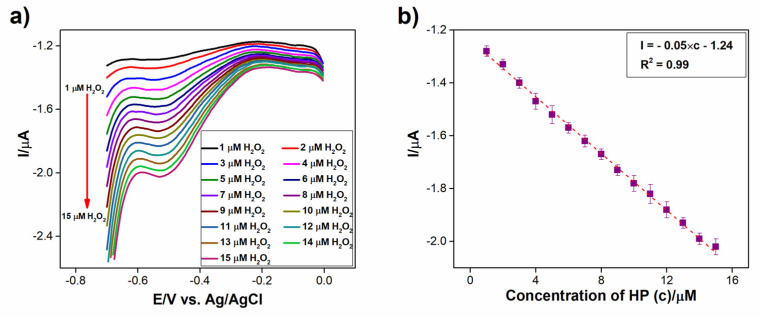
(**a**) DPVs obtained for the reduction of 1 µM of H_2_O_2_ at Co_3_S_4_/GCNNS modified GCE electrode in 0.2 M PBS of pH 7.2, (**b**) calibration plot of peak current against the concentration of H_2_O_2_.

As illustrated in Figure 10a, differential pulse voltammetry (DPV) was achieved for the successive injection of 1 µM of H_2_O_2_ in 0.2 M PBS (pH 7.2) at the Co_3_S_4_/GCNNS electrode. The reduction current rose linearly with each addition of H_2_O_2_. Still, the reduction potential remained constant at −0.52 V. This suggests that it is possible to sensitively measure H_2_O_2_ at Co_3_S_4_/GCNNS without impacting its reduction potential. The regression relation I = −0.05 × c − 1.24 represents the calibration plot between the reduction current and concentration of H_2_O_2_ in Figure 10b, and it displays a strong linear relationship with an R^2^ value of 0.99.

#### 3.3.3. Amperometric Sensing of H_2_O_2_ at Co_3_S_4_/GCNNS Modified GCE

**Figure 11:**
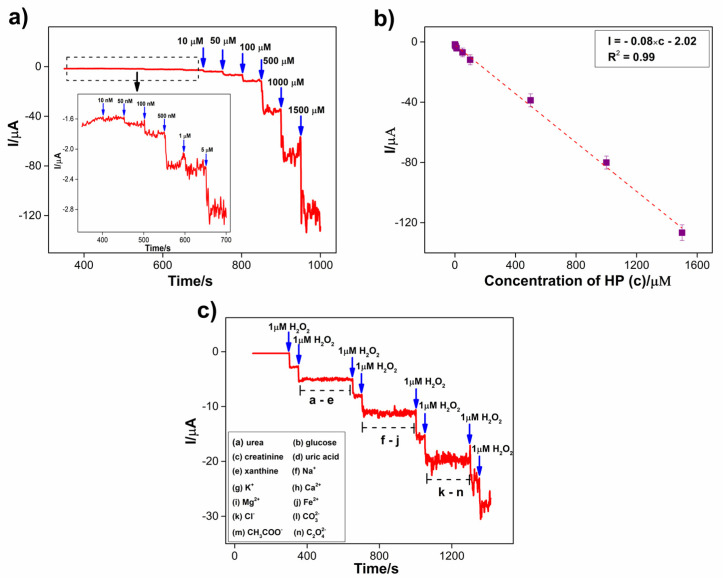
Amperometric i-t curve obtained for the reduction of H_2_O_2_ at Co_3_S_4_/GCNNS in 0.2 M PBS (pH 7.2) at an applied potential of −0.7 V for (**a**) an addition of H_2_O_2_ of concentration from 10 nM to 1.5 mM, (**b**) calibration plot of current vs. concentration of H_2_O_2_, (**c**) for the reduction of 1 µM H_2_O_2_ in presence of 500 µM of potential interferences.

Wide-range amperometry was employed to evaluate the application of the Co_3_S_4_/GCNNS electrode in the detection of H_2_O_2_, as depicted in Figure 11a. The amperometric i–t curve was obtained at a constant potential of −0.7 V, and the concentration of H_2_O_2_ varied from 10 nM to 1.5 mM in 0.2 M PBS of pH 7.2. During each addition of H_2_O_2_, a well-defined increase in the current was observed. The sharp increase in the current is due to the electroreduction of H_2_O_2_ at the Co_3_S_4_/GCNNS electrode. A steady state of the final reduction current was reached in 3 s, indicating the rapid response of the electrode to H_2_O_2_. The amperometric response also demonstrates the excellent electrocatalytic performance of the Co_3_S_4_/GCNNS composite electrode. The linear increase in the reduction current with the increasing H_2_O_2_ concentration validates its practical application as an H_2_O_2_ sensor. As shown in Figure 11b, the calibration curve for the sensor was plotted between the H_2_O_2_ concentrations and the reduction currents obtained. The regression equation for the calibration curve is obtained as I = −0.08 × c − 2.02 showing good linearity with an R^2^ value of 0.99. The limit of detection of the sensor was calculated to be 70 nM (S/N = 3), and it shows a sensitivity of 1.16 μAµM^−1^ cm^−2^ (slope/area). The analytical performance of Co_3_S_4_/GCNNS is compared to various non-enzymatic sensors in Table S2. Shu et al. developed a TiO_2_/SiO_2_ composite as a phosphorescence sensor for H_2_O_2_ [47]. Although it detects H_2_O_2_ over a wide range of analyte concentrations, the LOD is as low as 0.16 µM. The graphene-CdS electroluminescence sensor detects H_2_O_2_ in a range from 5 μM to 1 mM but has a low LOD of 1.7 μM [48]. Gan et al. explored MoS_2_ quantum dots as a fluorescence sensor and determined H_2_O_2_ in a narrow range of 2–20 µΜ [49]. Calorimetric detection of H_2_O_2_ has been demonstrated by porphyrin iron-grafted mesoporous silica composites, but the detection limit is low as 67 µΜ [50]. Ding et al. presented an optical sensor for H_2_O_2_ in the 1 µΜ to 10 mM range by growing Pt nanoparticles inside the pores of fibrous silica particles and with a low LOD of 15 µΜ [51]. Co_3_S_4_/GCNNS has a very high LOD and sensitivity than these sensors. Further, the performance of Co_3_S_4_/GCNNS is compared with related electrochemical sensors (Table S2). Co_3_O_4_ hollow-sphere-based H_2_O_2_ sensor has low LOD and sensitivity compared to Co_3_S_4_/GCNNS [52]. Au/Cu bimetallic nanoparticles reported by Gowthaman et al. are expensive and have low LOD and sensitivity [53]. Chen et al. presented Cu_2_S@Co_3_S_4_ for the excellent electrocatalytic reduction of H_2_O_2_ using CV but did not explore the sensor aspects in detail [24]. GCN hollow spheres operate in a short range and have low sensitivity and LOD [27]. Liu et al. synthesized ZnO/GCNNS for H_2_O_2_ oxidation, but the sensor has a low LOD [31]. Atacan et al. prepared CuO/GCN nanoflakes. They demonstrated the sensing of H_2_O_2_ using DPV with high sensitivity, but the operation is limited to a short range, and the LOD is in the micromolar range. Recently, Ye et al. reported ZnFe_2_O_4_/GCN nano-micro composite for H_2_O_2_ sensing but has a very low sensitivity and LOD compared to Co_3_S_4_/GCNNS [3]. It is visible that Co_3_S_4_/GCNNS is an improvement over these proposed H_2_O_2_ sensors.

One of the most common problems when using sensors to determine H_2_O_2_ in real samples is their response to the interfering species. To investigate the selectivity of Co_3_S_4_/GCNNS towards H_2_O_2_ detection, the influence of other common components in blood serum was examined by amperometry at −0.7 V in 0.2 M PBS of pH 7.2. It is reported that the H_2_O_2_ concentration present in human blood serum is generally less than 10 μM. In contrast, creatinine and xanthine are present in blood serum at concentrations below 119.3 μM and 2 mM, respectively. For non-diabetics, the normal glucose level is between 3.9 and 7.1 mM. The uric acid concentration ranges from 208 to 428 μM, and the urea concentration ranges between 2.5 and 7.5 mM [54,55,56,57]. On average, these species are present at less than or around 500 times the serum H_2_O_2_ concentration. Therefore, 500-fold concentrations of these potential interferences are chosen to study the selectivity of Co_3_S_4_/GCNNS towards the electroreduction of 1 μM H_2_O_2_. As shown in Figure 11c, after attaining a stable response in PBS, 1 µM H_2_O_2_ was added to the PBS, and the corresponding increase in the reduction current indicated the proper functioning of the Co_3_S_4_/GCNNS electrode. Then, 1 µM H_2_O_2_ was added in another step to obtain the same increase in reduction current as in the previous step. Further, every 60 s, 500 µM of urea, glucose, creatinine, uric acid, and xanthine were added, respectively, to the region marked as a–e (Figure 11c). As shown, no appreciable increase in the current was observed upon their addition, confirming that they show no interference at the Co_3_S_4_/GCNNS electrode. The amperometric current at the modified electrode increases for two consecutive additions of 1 μM of H_2_O_2_, indicating that the previously added species has no effect on the reduction of H_2_O_2_ at Co_3_S_4_/GCNNS. Furthermore, the influence of the common cations present in the blood serum was studied by adding 500 µM of salts of each Na^+^, K^+^, Ca^2+^, Mg^2+,^ and Fe^2+^, respectively (f–j); nevertheless, no change in current was observed, still a spike of 1 µM of H_2_O_2_ increases the current response indicating the selectivity of Co_3_S_4_/GCNNS electrode towards H_2_O_2_ than to these cations. Further 500 µM concentrations of anions, Cl^−^, CO_3_^2−^, CH_3_COO^−^, and C_2_O_4_^2−^ were each added at 60 s intervals, as shown in the region; k–n, and only a negligible change in current was observed, whereas two successive additions of H_2_O_2_ resulted in the increase of current due to the reduction of H_2_O_2_. The results confirm that 500-fold additions of these entities do not alter the current response due to H_2_O_2_ reduction, proving the high selectivity of the Co_3_S_4_/GCNNS electrode as an H_2_O_2_ sensor.

#### 3.3.4. Stability of Co_3_S_4_/GCNNS Modified GCE Sensor

**Figure 12:**
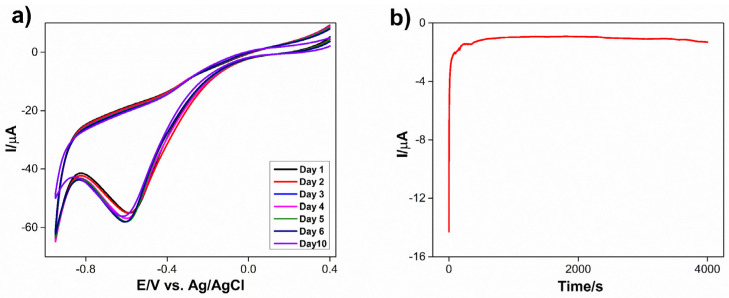
(**a**) CVs obtained for the reduction of 1 mM of H_2_O_2_ at Co_3_S_4_/GCNNS modified GCE in 0.2 M PBS for days from 1 to 10, (**b**) amperometric i–t curve obtained for Co_3_S_4_/GCNNS modified GCE for 4000s at an applied potential of −0.7 V in 0.2 M PBS pH 7.2.

Cyclic voltammetric responses of Co_3_S_4_/GCNNS-modified GCE towards 1 mM of H_2_O_2_ in 0.2 M PBS (pH = 7.2) were obtained for many 1, 2, 3, 4, 5, 6, and 10 days to confirm the long-term stability and repeatability of the proposed sensor, and the recorded cyclic voltammograms are shown in Figure 12a. The H_2_O_2_ reduction current showed no noticeable change, and the reduction potential remained unaltered, indicating the good stability of the fabricated sensor toward H_2_O_2_ reduction. Furthermore, the amperometric i–t curve of the Co_3_S_4_/GCNNS electrode was recorded for 4000 s at an applied potential of −0.7 V in 0.2 M PBS (pH 7.2) and is shown in Figure 12b. The current response was constant throughout the experiment, showing good amperometric stability of the Co_3_S_4_/GCNNS electrode in PBS.

#### 3.3.5. Real Sample Analysis

**Figure 13:**
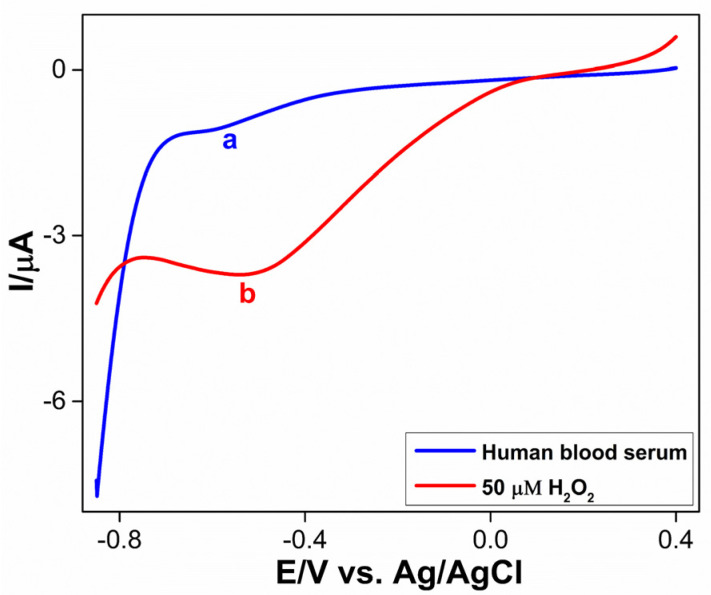
DPVs obtained for (**a**) human blood serum and (**b**) addition of 50 µM H_2_O_2_ at Co_3_S_4_/GCNNS modified GCE in serum.

The determination of H_2_O_2_ in human blood serum was performed at the Co_3_S_4_/GCNNS electrode using DPV in 0.2 M PBS of pH 7.2 to check the practical applicability of the proposed sensor, which is shown in Figure 13 (curve a). The reduction peak for H_2_O_2_ in the serum sample appears around −0.52 V. The H_2_O_2_ concentration in the serum was found to be 3.2 µM, which is within the normal range. An additional 50 µM of H_2_O_2_ was added to the serum, and the reduction peak current increased without affecting the reduction potential, as shown in Figure 13 (curve b). A recovery of 98.5 % was achieved. This indicates that the proposed sensor can be employed for monitoring H_2_O_2_ in real samples.

## 4. Conclusions

Co_3_S_4_/GCNNS were synthesized as a promising material for the electrocatalytic reduction of H_2_O_2_ by a simple and low-cost method. Physical and chemical characterizations helped us to confirm the successful formation of the materials. Nanosheets of GCNNS and micro flowers of Co_3_S_4_ were observed by SEM. From CV, the Co_3_S_4_/GCNNS composite was observed to reduce H_2_O_2_ and provide 4.7 and 7.1 times higher reduction current compared to pure Co_3_S_4_ and GCNNS, respectively, showing excellent electrocatalytic activity. The hybridization between Co_3_S_4_ and GCNNS enhanced the electroactive surface area and conductivity of the proposed sensor, which are crucial for superior electrocatalytic activity. At the same time, as evident from XPS studies, the electronic interactions between GCNNS and Co_3_S_4_ enhance the catalytically active redox centers on the Co_3_S_4_/GCNNS surface for H_2_O_2_ reduction. Co_3_S_4_/GCNNS was further explored for the amperometric sensing of H_2_O_2_ to show high performance over a wide range from 10 nM to 1.5 mM with a high detection limit of 70 nM. The sensor showed a fast response and excellent selectivity against potential interferences, and the practicality of the sensor was evaluated by the determination of H_2_O_2_ in the human serum. The current work demonstrates the potential of Co_3_S_4_/GCNNS as an ideal material for constructing high-performance H_2_O_2_ sensors.

## Data Availability

Data can be made available upon request.

## Supplementary Materials

The following supporting information can be downloaded at: https://www.mdpi.com/article/10.3390/bios13010108/s1, Details on materials, characterization, and electrochemical studies are provided. Figure S1: XPS spectra of GCNNS, (a) survey scan, high-resolution spectra of (b) C 1s and (c) N1s. XPS spectra of Co_3_S_4_, (d) survey scan, high-resolution spectra of (e) Co 2p and (f) S 2p., Figure S2: Comparison of PL spectra of Co_3_S_4_, GCNNS, and Co_3_S_4_/GCNNS measured at an excitation wavelength of 330 nm., Figure S3: (a) CVs obtained for the reduction of 1 mM H_2_O_2_ at 1 mg/mL GCNNS containing various amounts of Co_3_S_4_; 0.1, 0.15, 0.2, and 0.25 mg/mL in 0.2 M PBS pH 7.2, (b) CVs obtained for the reduction of 1 mM H_2_O_2_ at 0.2 mg/mL Co_3_S_4_ containing various amount of GCNNS of 0.5, 1 and 1.5 mg/mL. Table S1: Comparison of the binding energy of the core levels in Co_3_S_4_, GCN, and Co_3_S_4_/GCNNS. Table S2: Comparison of related non-enzymatic electrochemical sensors for H_2_O_2_ sensors with the performance of Co_3_S_4_/GCNNS.
