# Supplementary material for: Hybridization of Co3S4 and Graphitic Carbon Nitride Nanosheets for High-performance Nonenzymatic Sensing of H2O2
Source: Biosensors (Basel). 2023 Jan 7;13(1):108. doi: 10.3390/bios13010108 (PMC9856010; doi:10.3390/bios13010108)
Supplement: Supplementary file 1 [file biosensors-13-00108-s001.zip › biosensors-2083684-supplementary.pdf]

Supporting Information

Hybridization of Co_3S_4 and Graphitic Carbon Nitride Nanosheets for High-performance Nonenzymatic Sensing of H_2O_2

Asha Ramesh [1], Ajay Ajith [2], Neeraja Sinha Gudipati [1], Siva Rama Krishna Vanjari [3], S. Abraham John [2], Vasudevanpillai Biju [4], and Ch Subrahmanyam [1,*]

[1]Department of Chemistry, Indian Institute of Technology Hyderabad, Kandi, Sangareddy, 502285, Telangana, India
[2]Centre for Nanoscience and Nanotechnology, Department of Chemistry, The Gandhigram Rural Institute, Gandhigram, Dindigul, 624302, Tamilnadu, India
[3]Department of Electrical Engineering, Indian Institute of Technology Hyderabad, Kandi, Sangareddy, 502285, Telangana, India
[4]Research Institute for Electronic Science Hokkaido University, Sapporo, Hokkaido 001-0020, Japan
***** Correspondence: csubbu@chy.iith.ac.in

1. Materials

Cobalt(II) nitrate hexahydrate [$Co(NO_3)_2 \cdot 6H_2O$, Sigma-Aldrich, 99.5%], Thiourea [Merck, 99%], Ethylenediamine [Alfa Aesar, 99%], Melamine[Alfa Aesar, 99%]. All the chemicals were of analytical grade and used without further purification.

2. Characterization

Crystallographic properties of the as-prepared materials were characterized via Powder X-ray diffraction using a Rigaku SmartLab X-ray diffractometer with Cu Kα (λ=1.54 Å) radiation and a Ni filter over a Braggs angle of 10-80°. The chemical composition of the materials and oxidation states of the elements were examined by X-ray photoelectron spectroscopy (XPS) using AXIS Supra-Kratos analytical spectrophotometer with a monochromatic X-ray source, Al Kα, of 1486.6 eV. The morphological studies of the materials were performed by scanning electron microscopy (SEM) using VEGA3 TESCAN, Czech Republic. Elemental analysis of the samples was further performed by energy-dispersive X-ray spectroscopy (EDS) during SEM characterization. JEOL JEM F200 transmission electron microscope was used to record TEM images and SAED patterns of the materials at an acceleration voltage of 200 kV. Absorption studies were performed in UV-Vis spectrophotometer (T90+, PG Instruments). Photoluminescence (PL) studies were performed using HORIBA Scientific FluoroMax-4 spectrofluorometer.

3. Electrochemical studies

Electrochemical studies were carried out using a three-electrode configuration in CHI electrochemical workstation (Model 643B, Austin, TX). The three electrodes used are a material-coated glassy carbon electrode as the working, a Pt wire as the counter, and

Ag/AgCl as the reference electrode. A phosphate buffer solution (PBS) of pH 7.2 was used as an electrolyte for the electrochemical studies. Cyclic voltammograms are recorded after deaerating the solution with nitrogen purging. The behavior towards the Fe^{3+}/Fe^{2+} couple and the electrochemical impedance spectra of the modified electrodes were recorded in 0.1 M KCl containing 1mM of $K_3(Fe(CN)_6$ and 1mM of $K_4Fe(CN)_6$.

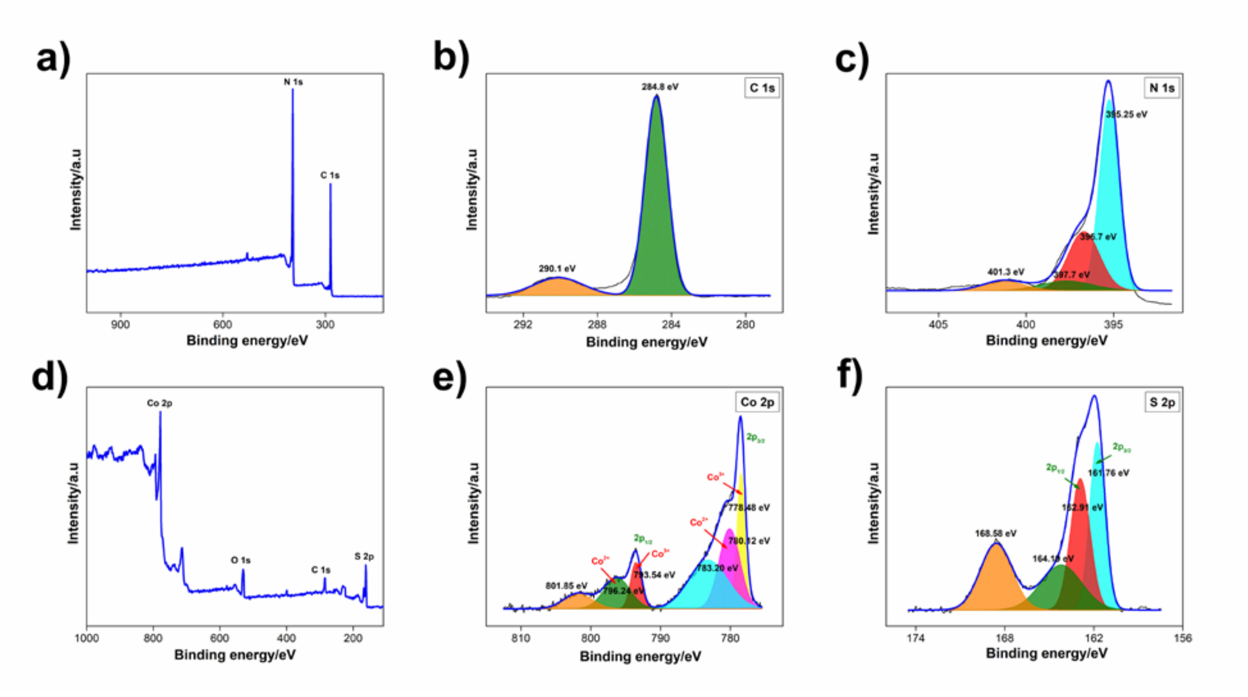

Figure S1. XPS spectra of GCNNS, (**a**) survey scan, high-resolution spectra of (**b**) C 1s and (**c**) N1s. XPS spectra of Co_3S_4, (**d**) survey scan, high-resolution spectra of (**e**) Co 2p and (**f**) S 2p.

Table S1. Comparison of the binding energy of the core levels in Co_3S_4, GCNNS, and Co_3S_4/GCNNS.

Core level	Material	Binding Energy (eV)
C 1s	GCNNS	284.8, 290.1
	Co_3S_4/GCNNS	284.8, 288.0
N 1s	GCNNS	395.25, 396.7, 397.7, 401.3
	Co_3S_4/GCNNS	398.4, 399.3, 400.5, 404.6
Co 2p	Co_3S_4	778.48, 793.54, 780.12, 796.24, 783.20, 801.85
	Co_3S_4/GCNNS	783.3, 798.26, 780.7, 796.1, 804.2
	Co_3S_4	161.76, 162.91, 164.19, 168.58

S 2p	Co_3S_4/GCNNS	161.9, 164.9, 166.8, 171.48

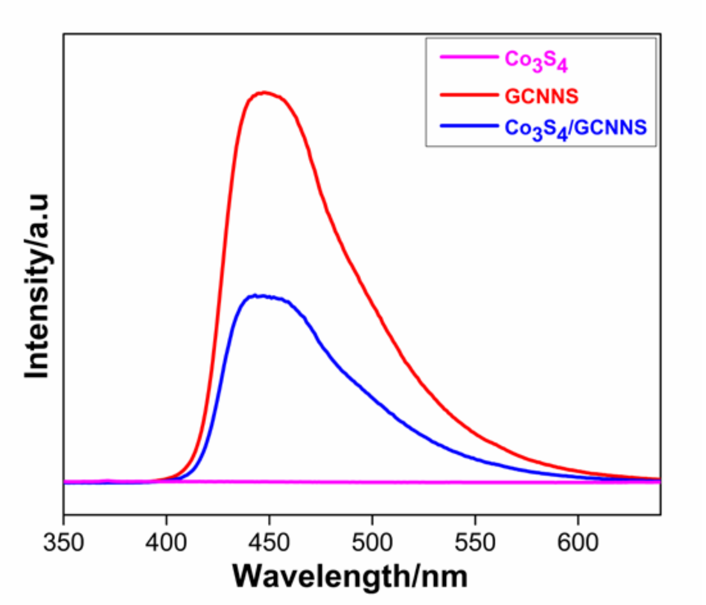

Figure S2. Comparison of PL spectra of Co_3S_4, GCNNS, and Co_3S_4/GCNNS measured at an excitation wavelength of 330 nm.

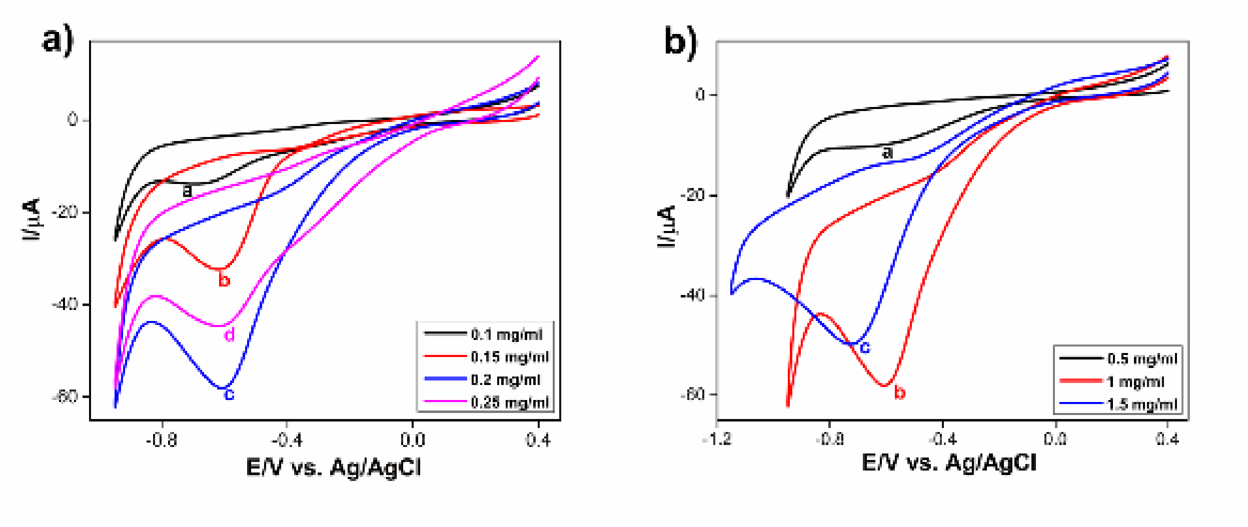

Figure S3. (**a**) CVs obtained for the reduction of 1 mM H_2O_2 at 1 mg/mL GCNNS containing various amounts of Co_3S_4; 0.1, 0.15, 0.2, and 0.25 mg/mL in 0.2 M PBS of pH 7.2, (**b**) CVs obtained for the reduction of 1 mM H_2O_2 at 0.2 mg/mL Co_3S_4 containing various amount of GCNNS of 0.5, 1 and 1.5 mg/mL in 0.2 M PBS of pH 7.2.

Table S2. Comparison of related non-enzymatic H_2O_2 sensors with the performance of Co_3S_4/GCNNS.

Material	Technique	Linear range (μM)	LOD (μM)	Sensitivity ($\mu A \mu M^{-1} cm^{-2}$)	Reference
TiO_2/SiO_2	Phosphorescence	7 - 70000	0.16	-	[1]
Graphene-CdS	Electrochemiluminescence	5 - 1000	1.7	-	[2]
MoS_2	Fluorescence	2 - 20	-	-	[3]
Porphyrin Iron-grafted mesoporous silica	Colorimetry	293 - 8800	67	-	[4]
Pt NPs@fibrous silica	Optical sensor	1 - 10000	15	-	[5]
Co_3O_4	Amperometry	0.4 - 2200	0.105	0.96	[6]
Au/Cu	Amperometry	20 - 100	0.073	0.94	[7]
$Cu_2S@Co_3S_4$	CV	-	-	-	[8]
GCN hollow spheres	CV	0.150 - 1.8	26.48	0.35	[9]
ZnO/GCN	Chronoamperometry	5 - 200	2.0	-	[10]
CuO/GCN	DPV	0.5 - 50	0.31	3.327	[11]
$ZnFe_2O_4$/GCN	Amperometry	5 - 200	1	0.19	[12]
Co_3s_4/GCNNS	Amperometry	0.01 - 1500	0.070	1.160	This work

References

1. Shu, X.; Chen, Y.; Yuan, H.; Gao, S.; Xiao, D. H_2O_2 Sensor Based on the Room-Temperature Phosphorescence of Nano TiO_2 /SiO_2 Composite. *Anal. Chem.* **2007**, *79*, 3695–3702. https://doi.org/10.1021/ac0624142.

2. Wang, K.; Liu, Q.; Wu, X.-Y.; Guan, Q.-M.; Li, H.-N. Graphene enhanced electrochemiluminescence of CdS nanocrystal for H_2O_2 sensing. *Talanta* **2010**, *82*, 372–376. https://doi.org/10.1016/j.talanta.2010.04.054.

3. Gan, Z.; Gui, Q.; Shan, Y.; Pan, P.; Zhang, N.; Zhang, L. Photoluminescence of MoS_2 quantum dots quenched by hydrogen peroxide: A fluorescent sensor for hydrogen peroxide. *J. Appl. Phys.* **2016**, *120*, 104503. https://doi.org/10.1063/1.4962318.

4. Zhu, P.; Xu, Z.; Cai, L.; Chen, J. Porphyrin Iron-Grafted Mesoporous Silica Composites for Drug Delivery, Dye Degradation and Colorimetric Detection of Hydrogen Peroxide. *Nanoscale Res. Lett.* **2021**, *16*, 41. https://doi.org/10.1186/s11671-021-03501-6.
5. Ding, L.; Chen, S.; Zhang, W.; Zhang, Y.; Wang, X. Fully Reversible Optical Sensor for Hydrogen Peroxide with Fast Response. *Anal. Chem.* **2018**, *90*, 7544–7551. https://doi.org/10.1021/acs.analchem.8b01159.
6. Wang, M.; Jiang, X.; Liu, J.; Guo, H.; Liu, C. Highly sensitive H_2O_2 sensor based on Co_3O_4 hollow sphere prepared via a template-free method. *Electrochim. Acta* **2015**, *182*, 613–620. https://doi.org/10.1016/j.electacta.2015.08.116.
7. Gowthaman, N.; Shankar, S.; John, S.A. Substrate catalyzed formation of Au-Cu bimetallic nanoparticles as electrocatalyst for the reduction of dioxygen and hydrogen peroxide. *J. Electroanal. Chem.* **2018**, *812*, 37–44. https://doi.org/10.1016/j.jelechem.2018.01.053.
8. Chen, H.; Cai, J.; Yang, J.; Zhong, Z.; Ma, M.; Deng, W.; Tan, Y.; Xie, Q. Synergistic electrocatalysis of $Cu_2S@Co_3S_4$ core-shell heterostructures toward H_2O_2 reduction and their application for sensitive immunosensing of alpha fetoprotein. *Sens. Actuators B Chem.* **2021**, *348*, 130703. https://doi.org/10.1016/j.snb.2021.130703.
9. Puentes-Prado, E.; Gutiérrez-Granados, S.; Gómez-Solís, C.; Ramírez-García, G. Enhanced photo-electrochemical response of screen-printed electrodes based on g-C_3N_4 hollow spheres and other morphologies for sensing applications. *FlatChem* **2022**, *35*, 100412. https://doi.org/10.1016/j.flatc.2022.100412.
10. Liu, H.; Zhang, Y.; Dong, Y.; Chu, X. Synthesis of ZnO/g-C_3N_4 Nanocomposite and Its Electrochemical Application in Hydrogen Peroxide Detection. *Russ. J. Electrochem.* **2021**, *57*, 808–815. https://doi.org/10.1134/S1023193520120125.
11. Atacan, K.; Özacar, M. Construction of a non-enzymatic electrochemical sensor based on CuO/g-C_3N_4 composite for selective detection of hydrogen peroxide. *Mater. Chem. Phys.* **2021**, *266*, 124527. https://doi.org/10.1016/j.matchemphys.2021.124527.
12. Ye, M.; Yang, C.; Sun, Y.; Wang, J.; Wang, D.; Zhao, Y.; Zhu, Z.; Liu, P.; Zhu, J.; Li, C.; et al. $ZnFe_2O_4$ /Graphitic Carbon Nitride Nano/Microcomposites for the Enhanced Electrochemical Sensing of H_2O_2. *ACS Appl. Nano Mater.* **2022**, *5*, 10922–10932. https://doi.org/10.1021/acsanm.2c02204.
